# Non-human Primate Models for Brain Disorders – Towards Genetic Manipulations *via* Innovative Technology

**DOI:** 10.1007/s12264-017-0115-4

**Published:** 2017-03-01

**Authors:** Zilong Qiu, Xiao Li

**Affiliations:** Institute of Neuroscience, Key Laboratory of Primate Neurobiology, State Key Laboratory of Neuroscience, Center for Excellence in Brain Science and Intelligence Technology, Shanghai Institute for Biological Sciences, Chinese Academy of Sciences, Shanghai, 200031 China

**Keywords:** Non-human primates, Brain disorders, Genome editing, Autism, Neurological disorders, Psychiatric disorders

## Abstract

Modeling brain disorders has always been one of the key tasks in neurobiological studies. A wide range of organisms including worms, fruit flies, zebrafish, and rodents have been used for modeling brain disorders. However, whether complicated neurological and psychiatric symptoms can be faithfully mimicked in animals is still debatable. In this review, we discuss key findings using non-human primates to address the neural mechanisms underlying stress and anxiety behaviors, as well as technical advances for establishing genetically-engineered non-human primate models of autism spectrum disorders and other disorders. Considering the close evolutionary connections and similarity of brain structures between non-human primates and humans, together with the rapid progress in genome-editing technology, non-human primates will be indispensable for pathophysiological studies and exploring potential therapeutic methods for treating brain disorders.

## Introduction

Psychiatric disorders are a group of mental illnesses without detectable pathological symptoms such as neuronal death. They include schizophrenia, depression, bipolar disorder, and autism spectrum disorders (ASDs). The onset of most psychiatric disorders is normally in adulthood. However, the onset of ASDs is during the first 3 years of life. Therefore, ASDs are also considered to be developmental disorders, possibly caused by disruption of neural development leading to abnormal social behaviors and other symptoms [1].

With recent advances in human genetics and neurobiological studies, ASDs have been found to include both genetic and environmental components. Particularly, mechanistic studies in animal models have provided tremendous insights into how genetic and environmental factors contribute to neural development and underlie the proper functioning of cognitive and social behaviors. A wide range of animal models including worms, fruit flies, zebrafish, and rodents have been used to study neural development and disorders. However, whether complicated neurological and psychiatric symptoms can be faithfully mimicked in animals other than primates is still debatable. In this review, we discuss technical advances for studying the neural mechanisms underlying ASDs and other brain disorders using non-human primates as animal models. Considering the close evolutionary connections and similarity of brain structures in non-human primates and humans, together with the rapid progress of genome-editing technology, non-human primates will be indispensable in pathophysiological studies and potential interventions for psychiatric disorders [2].

## Pharmacological and Environmental Approaches

The general approaches used in rodents for modeling psychiatric disorders, such as chronic restraint stress, chronic unpredictable mild stress, and chronic social defeat stress, are efficient for inducing anxiety-like behaviors for various periods of time. However, for ethical reasons, most of these approaches are not practical in non-human primates. Under these circumstances, researchers have designed mild stress models, in which monkeys are subjected to gentle stresses. Kalin and colleagues have established various stress models in monkeys, followed by assessment of physiological and neurochemical parameters [3]. After being stared by humans, infant monkeys appear to exhibit anxiety-like activity such as aggressive barking. Infant monkeys make distress calls when separated from their mothers. Investigators then found, using pharmacological approaches, that the opiate and GABA systems play different roles in regulating these behaviors, suggesting roles of these neuromodulators in emotional behaviors in primates [3].

With regard to the close relationships between monkeys and humans, whether complex environmental factors can be applied to monkeys is a critical question to address. Hu and colleagues have designed a paradigm in which maternal separation paradigms are mimicked in monkeys [4]. They investigated the impact of maternal separation in early life on social behaviors during adolescence, as well as levels of stress hormones. Importantly, they found that maternal separation during early life dramatically influences social behaviors and stress levels, consistent with previous studies in humans. Further mechanistic studies using these monkeys may elucidate the underlying circuit basis of how early life stress affects behaviors during adulthood. The classical methods of studying the neural mechanisms underlying psychiatric disorders have been nicely summarized by Suomi [5] and Watson and Platt [6].

## Genetic Approaches Based on Viral Transduction

Over the years, genetic approaches have been widely applied in model organisms, such as worms, fruit flies, and rodents, to establish models of brain disorders. Due to the difficulty of handling germline cells and the unavailability of embryonic stem cells, genetic engineering in monkeys did not become available until 2001 [7]. In this seminal work, Chan and colleagues infected rhesus monkey (*Macaca mulatta*) oocytes with a retrovirus harboring the green fluorescence protein (GFP) gene, followed by *in vitro* fertilization (IVF) with sperm. After transplantation of fertilized eggs into surrogate monkeys, researchers successfully acquired transgenic monkeys carrying the GFP gene. This pioneering work launched a new era of genetic engineering in non-human primates.

Taking advantage of the high efficiency of lentiviral infection, Li, Chan, and colleagues at Emory University used lentivirus harboring the disease-causing Huntingtin (HTT) mutant gene to infect rhesus monkey oocytes [8]. After IVF, investigators successfully obtained several transgenic monkeys carrying the HTT mutant gene. Remarkably, the monkeys carrying the mutant gene showed clear signs of neuronal death in the brain, which is barely detectable in rodent models of Huntington disease. Thus, for the first time, a non-human primate model of a human brain disorder exhibited the unique advantage of mimicking the neuropathological symptoms, which are difficult to find in rodent models.

Another pioneering work in genetic engineering for non-human primates was done in marmosets (*Callithrix jacchus*). Sasaki and colleagues also used lentiviral-based methods to produce transgenic marmosets carrying GFP transgenes [9]. Importantly, they obtained a second generation carrying the transgene, taking advantage of the shorter reproduction in marmosets than macaques, and indicating the reliability of transgenic monkeys as animal models.

Given the availability of lentiviral transduction methods, a series of transgenic monkeys have been produced during the last several years to model various brain disorders. In order to make monkey models of Parkinson disease, Li and colleagues produced transgenic monkeys expressing the mutant form of α-synuclein (A53T) [10]. Although the α-synuclein (A53T) transgenic monkeys have not yet shown Parkinsonism-like symptoms, such as tremor and degeneration of dopaminergic neurons, various cognitive defects and neuropathology in the brain have been identified, suggesting that α-synuclein (A53T) causes pathological changes in the monkey brain [11].

Due to the long reproductive cycle of macaques (typically reaching sexual maturation at 4–5 years of age), obtaining a second generation is time-consuming and costly. In recent work on making monkey models of ASDs, Qiu and colleagues used lentivirus-based transgenic methods to produce transgenic monkeys with the autism gene *MECP2* and examined social and motor behaviors associated with social interaction and cognition. They found that *MECP2* transgenic monkeys indeed exhibit abnormal behaviors, including repetitive locomotor activity, elevated anxiety levels, and defects in social interactions, which nicely mimic ASDs in human patients [12]. Remarkably, investigators applied xenograft methods to transplant testicular tissues from one sexually immature transgenic monkey into nude mice. After 9 months, the testicular tissues became mature and the mature sperm were collected for IVF. Second-generation transgenic monkeys were obtained ~3.5 years after the birth of the first-generation transgenic monkeys, dramatically shortening the time for acquiring a second generation [12]. This work provides a framework in which monkey models of brain disorders can be established.

## Genetic Approaches Based on Genome-Editing Methods

With the emergence of the CRISPR-Cas9 and TALEN genome-editing methods from 2011, genetic engineering in non-human primates became feasible and relatively easy. The landmark work was done by Niu and colleagues, who injected Cas9 mRNA along with single-guide RNA (sgRNA) into the fertilized eggs of cynomolgus monkeys (*Macaca fascicularis*) and transplanted them into surrogate monkeys [13]. Among the newborn monkeys, researchers identified genomic mutations in the sgRNA-targeting sites and the cellular effects of genetic deletions. However, due to the prolonged effective period of CRISPR/Cas9, the mutant monkeys are normally mosaics with various mutations, which also differ across tissues. The mosaicism in the offspring monkeys interferes with the interpretation of the consequences of genetic deletions caused by CRISPR/Cas9.

In another study, Chen and colleagues used CRISPR/Cas9 to target the dystrophin gene, mutation of which leads to Duchenne muscular dystrophy (DMD) [14]. Although the genetic mutation was clearly identified in sgRNA-targeted sites of the monkey DMD gene, whether the DMD mutation leads to behavioral abnormalities in monkeys is yet to be determined.

To model Rett syndrome, a developmental disorder, investigators used TALEN-based genome-editing to target the *MECP2* (methyl-CpG binding protein 2) gene in cynomolgus monkeys [15, 16], and reported that TALEN is also effective in inducing genomic mutations in the monkey genome and yield offspring carrying mutations in the *MECP2* gene. However, whether genomic mutation of *MECP2* leads to behavioral changes similar to human Rett syndrome remains to be determined [15].

## Connectomic View of the Psychiatric Brain

The rapid progress of genetic engineering in non-human primates provides researchers with a wide platform to study primate brains at an unimaginable scale. It is likely that brain imaging and cognitive studies using genetically-engineered monkeys will be available soon, and will provide causal links between genes and brain structure in the non-human primate. An elegant example is the work by Wang and colleagues, who investigated the effects of ketamine on the functional brain network using magnetic resonance imaging [17].

Although ketamine, a non-competitive N-methyl-D-aspartate receptor antagonist, has rapid and prolonged antidepressant effects in depressed patients after a single dose, the underlying mechanisms by which ketamine affects brain structure in patients are unknown. Given the lack of models of depression in non-human primates, researchers took another approach by giving rhesus monkeys a single dose of ketamine, and examining the functional connectivity of the brain using magnetic resonance imaging. They found that ketamine treatment leads to large-scale downregulation of functional connectivity, which is precisely opposite to the brain network in depressed patients, suggesting that ketamine has antidepressant effects by affecting the global brain network. This work remarkably illustrates the value of cutting-edge technology such as brain imaging in studying the neural mechanisms underlying brain disorders.

## Concluding Remarks

We are in an era when multiple disciplines have interacted to elucidate the mechanisms of complicated human brain disorders. Given the complexity of the brain, it has always been difficult to recapitulate the core symptoms of human brain disorders in animal models. With the rapid development of genetic-engineering technology, such as lentiviral transduction and genome-editing, it is foreseen that non-human primates will play a crucial role as animal models in the near future. It is worthwhile noting that current therapeutic methods for human brain disorders such as deep brain stimulation and transcranial magnetic stimulation will also need to be tested in non-human primates prior to human trials. Therefore, non-human primate models of brain disorders are also indispensable for translational studies. Together with the technical advances in brain imaging and non-invasive neural modulation methods such as ultrasonic stimulation, non-human primates will play more competitive roles in modeling brain disorders and serve as a platform on which researchers can seek potential therapies.
